# Irisin Recovers Osteoarthritic Chondrocytes In Vitro

**DOI:** 10.3390/cells9061478

**Published:** 2020-06-17

**Authors:** Gianluca Vadalà, Giuseppina Di Giacomo, Luca Ambrosio, Francesca Cannata, Claudia Cicione, Rocco Papalia, Vincenzo Denaro

**Affiliations:** 1 Laboratory of Regenerative Orthopaedics, Department of Orthopaedic and Trauma Surgery, Campus Bio-Medico University of Rome, Via Alvaro del Portillo 200, 00128 Rome, Italy; g.vadala@unicampus.it (G.V.); g.digiacomo@unicampus.it (G.D.G.); c.cicione@unicampus.it (C.C.); r.papalia@unicampus.it (R.P.); denaro@unicampus.it (V.D.); 2 Department of Endocrinology and Diabetes, Campus Bio-Medico University of Rome, Via Alvaro del Portillo 200, 00128 Rome, Italy; f.cannata@unicampus.it

**Keywords:** irisin, osteoarthritis, cartilage, physical exercise, chondrocyte

## Abstract

Physical exercise favors weight loss and ameliorates articular pain and function in patients suffering from osteoarthritis. Irisin, a myokine released upon muscle contraction, has demonstrated to yield anabolic effects on different cell types. This study aimed to investigate the effect of irisin on human osteoarthritic chondrocytes (hOAC) in vitro. Our hypothesis was that irisin would improve hOAC metabolism and proliferation. Cells were cultured in growing media and then exposed to either phosphate-buffered saline (control group) or human recombinant irisin (experimental group). Cell proliferation, glycosaminoglycan content, type II/X collagen gene expression and protein quantification as well as p38/extracellular signal-regulated kinase (ERK) mitogen-activated protein kinase (MAPK), protein kinase B (Akt), c-Jun N-terminal kinase (JNK), and nuclear factor kappa-light-chain-enhancer of activated B cells (NFκB) involvement were evaluated. Furthermore, gene expression of interleukin (IL)-1 and -6, matrix metalloproteinase (MMP)-1 and -13, inducible nitric oxide synthase (iNOS), and tissue inhibitor of matrix metalloproteinases (TIMP)-1 and -3 were investigated following irisin exposure. Irisin increased hOAC cell content and both type II collagen gene expression and protein levels, while decreased type X collagen gene expression and protein levels. Moreover, irisin decreased IL-1, IL-6, MMP-1, MMP-13 and iNOS gene expression, while increased TIMP-1 and TIMP-3 levels. These effects seemed to be mediated by inhibition of p38, Akt, JNK and NFκB signaling pathways. The present study suggested that irisin may stimulate hOAC proliferation and anabolism inhibiting catabolism through p38, Akt, JNK, and NFκB inactivation in vitro, demonstrating the existence of a cross-talk between muscle and cartilage.

## 1. Introduction

Osteoarthritis (OA) is a degenerative joint disorder affecting more than 10% of adults older than 60 years of age. It is characterized by increasing joint pain and stiffness, often leading to disability, with a tremendous negative impact on patients’ overall functionality and quality of life, as well as on healthcare expenditure [1]. Indeed, it has been estimated that the average cost for treating OA alone in the United States is higher than the combined cost for treating hypertension and diabetes [2].

Predominant features are articular cartilage damage and thinning, which are associated with chondrocyte hypertrophy, tissue inflammation, and extracellular matrix (ECM) degradation [3,4,5,6,7]. Major risk factors for OA include genetic predisposition, female gender, joint injury, and obesity [8]. Apart from mechanical overloading, obesity appears to further impact on OA pathogenesis through the secretion of proinflammatory adipokines involved in cartilage degradation, synovial inflammation and osteophytes development [9]. Weight loss and an active lifestyle are essential to reduce the risk of developing OA [10] and improve joint pain and stiffness in patients already affected with knee [11,12] and hip OA [13].

Irisin is a myokine that is secreted into the serum by skeletal muscle after physical exercise [14]. It was early recognized for its effects on glucose and fat metabolism, favoring thermogenesis and raising energy expenditure [15]. These pleiotropic effects could explain the benefits of muscle training in numerous metabolic disorders including obesity, metabolic syndrome, and diabetes [16,17]. Palermo et al. recently showed that osteoporotic fractures were associated with lower irisin serum levels, independently of other factors [18]. These data were supported by in vitro studies demonstrating that irisin can directly target osteoblasts and promote cell proliferation, differentiation, and matrix mineralization via the p38 mitogen-activated protein kinase (p38 MAPK) and extracellular signal-regulated kinase (ERK) signaling pathways [19]. Therefore, increasing evidence supports the role of skeletal muscle as an endocrine organ capable of secreting a wide range of myokines which communicate with other tissues and organs [20]. In this regard, irisin may act as a messenger between muscle and bone during physical exercise [21].

We hypothesized that irisin might maintain cartilage homeostasis through physical exercise acting as a cross-talk mechanism between muscle and cartilage. This novel idea is supported by a recent study reporting that serum and synovial fluid levels of irisin are negatively correlated with the severity of knee OA [22]. In this study, we isolated human osteoarthritic chondrocytes (hOAC) from specimens obtained during total knee replacement procedures. The hOAC were then cultured in presence of either recombinant irisin (r-irisin) or Dulbecco’s phosphate-buffered saline (DPBS) and evaluated for cell proliferation, glycosaminoglycan (GAG) production, type II and X collagen gene expression and protein synthesis. Furthermore, gene expression of inflammatory cytokines including interleukin (IL)-1 and -6, anti-catabolic enzymes such as tissue inhibitor of metalloproteinases (TIMP)-1 and -3 and catabolic markers including inducible nitric oxide synthase (iNOS), matrix metalloproteinase (MMP)-1 and -13 were investigated. Additional analyses were performed to assess p38, ERK, protein kinase B (Akt), and c-Jun N-terminal kinase (JNK) pathways involvement along with irisin effect on the downstream nuclear factor kappa-light-chain-enhancer of activated B cells (NFκB).

## 2. Materials and Methods

### 2.1. Cell Isolation 

The study was conducted in accordance with the Declaration of Helsinki, and the protocol was approved by the Ethics Committee of Campus Bio-Medico University of Rome. The hOAC were isolated from osteochondral tissues of eight patients (*n* = 8, Table 1) undergoing elective total knee joint replacement due to primary late-stage OA of the knee. Patients affected by rheumatoid arthritis or other forms of secondary OA, diabetes mellitus, systemic diseases as well as individuals who received intraarticular or systemic corticosteroids during the previous 3 months were excluded. Informed consent was obtained from each subject. The age of the patients ranged from 49 to 85 years and knee OA severity was assessed using the Kellgren–Lawrence (K-L) classification (grades 3 and 4). The hOAC were isolated according to a standardized procedure [23]. Specimens were minced and digested for 90 min at 37 °C with gentle agitation in sterile Dulbecco’s Modification of Eagle’s Medium (DMEM; Corning, Corning, NY, USA) containing 1% penicillin/streptomycin (P/S; Sigma, St. Louis, MO, USA), 5% fetal bovine serum (FBS; Corning, Corning, NY, USA), and 0.2% pronase (Calbiochem, San Diego, CA, USA). The remaining tissue was washed and digested overnight in DMEM with 1% P/S, 5% FBS, and 0.02% collagenase type II (Worthington, Lakewood, NJ, USA). The digest was filtered through a 70-µm pore size nylon mesh, the cells washed, resuspended in DMEM with 10% FBS and 1% P/S, and incubated at 37 °C in a humidified atmosphere of 5% CO_2_. The culture media were changed twice weekly and cultures were allowed to grow until reaching 80–90% confluence. Passage 1-hOAC were used for the experiments.

### 2.2. Dose-Response Relationships 

Micromasses were obtained as previously described [24]. Briefly, adherent cells were treated with trypsin-EDTA (Corning, Corning, NY, USA) and washed. 2.5 × 10^5^ hOAC were resuspended in culture media and centrifuged at low speeds (2000× g) for 5 min to form aggregates. After 24 h, cell aggregates were detached from the bottom of the tube and the media were changed to begin the treatments. Micromasses were treated either with DPBS (Euroclone, Pero, Italy) (Ctrl) or r-irisin (Sigma, St. Louis, MO, USA) for 7 days at a concentration of 25, 50, 75, and 100 ng/mL, which is the range between intraarticular [22] and blood concentrations in humans [19,25]. Media were changed three times during the week of culture. At the end of the experiment, micromasses were directly used to assess GAG content normalized to DNA as following. Micromasses were washed with PBS and digested with 100 µL of papain (Sigma, St. Louis, MO, USA) solution (0.25 mg/mL in 50 mM phosphate buffer, pH 6.5 containing 5 mM cysteine–hydrochloride and 5 mM ethylenediaminetetraacetic acid) overnight with gentle shaking at 65 °C. GAGs were measured by reaction with 1,9-dimethylmethylene blue (DMMB; Polysciences, Warrington, PA, USA) using chondroitin sulfate (Sigma, St. Louis, MO, USA) as a standard. Measurements of absorption were performed at a wavelength of 530 nm (Tecan Infinite M200 PRO). 

DNA content was assessed using PicoGreen Assay (Invitrogen, Carlsbad, CA, USA) as described by the manufacturer’s guidelines on cells extracts. A standard curve based on known concentration of DNA was used to determine the DNA content. The sample fluorescence was measured using a microplate reader (Tecan Infinite M200 PRO) at 460 nm and 540 nm wavelengths respectively. Data were expressed as quantity of GAG normalized to DNA content of chondrocytes cultured in growing media for 7 days, comparing the percent variation between the control group and the experimental group.

### 2.3. Cell Content 

Micromasses were treated either with DPBS (Ctrl) or r-irisin (Sigma, St. Louis, MO, USA) at a concentration of 25 ng/mL (concentration chosen after dose-response assay) for 14 days. Cell content was assessed by measuring DNA content using PicoGreen assay as described above at 4, 10, and 14 days of culture, according to previous reports [7]. A standard curve based on known DNA concentration was utilized to determine the DNA content. The assay was performed in triplicate for each donor.

### 2.4. RNA Extraction and Gene Expression Analysis 

Micromasses were treated either with DPBS (Ctrl) or recombinant irisin (r-irisin; Sigma, St. Louis, MO, USA) at a concentration of 25 ng/mL. Total RNA was extracted from pellets after 7 days of culture using the TRIzol reagent (Invitrogen, Carlsbad, CA, USA) according to the manufacturer’s instructions. cDNA was produced using the High Capacity cDNA Reverse Transcription kit (Applied Biosystems, Foster City, CA, USA) according to the manufacturer’s instructions. The mRNA levels were measured through qRT-PCR using TaqMan Gene Expression Assays and TaqMan Universal Master Mix II with UNG-Real Time PCR System Instrument 7900HT FAST according to manufacturer’s instructions. Gene expression assays collagen X (Hs00166657), collagen 2A1 (Hs00264051), IL-1β (Hs00174097), IL-6 (Hs00174131), TIMP-1 (Hs01092512), TIMP-3 (Hs00165949), iNOS (Hs01075529), MMP-1 (Hs00899658), MMP-13 (Hs00233992), and glyceraldehyde 3-phosphate dehydrogenase (GAPDH) (Hs03929097) were used. The expression level of each gene has been normalized to the expression of GAPDH and calculated as 2^-ΔΔCt^. Values in the experimental group were normalized to expression levels encountered in the control group, which was considered as a baseline. Reagents were purchased from Applied Biosystems (Foster City, CA, USA).

### 2.5. Protein Extraction and Western Blot Analysis

Micromasses were treated with r-irisin (25 ng/mL) or PBS (Ctrl) either for 7 days or at 10 min, 20 min, and 1 h time points. Subsequently, protein extraction and Western Blot analyses were performed. Cell lysates were obtained using radioimmunoprecipitation assay buffer (RIPA buffer; Sigma) for 30 min on ice, cleared by centrifugation for 30 min at 12000 g at 4 °C for 30 min and quantified using detergent compatible (DC) protein assay kit (Bio-Rad, Hercules, CA, USA). Total protein extracts (20 µg) from each sample were loaded on 4-12% SDS-PAGE gels, transferred onto nitrocellulose membranes through the Trans-Blot Turbo Transfer System (Bio-Rad, Hercules, CA, USA) and incubated in a blocking buffer (TBST 1X with 5% non-fat dry milk) for one hour. Membranes were incubated with primary antibody overnight shaking at 4 °C in TBST 1X with 1% non-fat dry milk. Anti-p38 (rabbit, 1:1000, Cell Signaling, Danvers, MA, USA), anti-phospho-p38 Thr180/Tyr182 (rabbit, 1:1000, Cell Signaling, Danvers, MA, USA), anti-p44/42 ERK1/2 (rabbit, 1:1000, Cell Signaling, Danvers, MA, USA), anti-phospho-p44/42 ERK1/2 Thr202/Tyr204 (rabbit, 1:2000, Cell Signaling, Danvers, MA, USA), anti-Akt (rabbit, 1:1000, Cell Signaling, Danvers, MA, USA), anti-phospho-Akt Ser473 (rabbit, 1:1000, Cell Signaling, Danvers, MA, USA), anti-Coll2A1 (mouse, 1:500, Novus Biologicals, Centennials, CO, USA), anti-Coll X (rabbit, 1:300, Abcam, Cambridge, UK), anti-SAPK/JNK (rabbit 1:1000, Cell Signaling, Danvers, MA, USA), anti-phospho-SAPK/JNK Tyr185 (mouse, 1:2000, Cell Signaling, Danvers, MA, USA), anti-NF-κB/p65 (rabbit 1:1000, Cell Signaling, Danvers, MA, USA), anti-phospho-NF-κB/p65 Ser536 (rabbit, 1:1000, Cell Signaling, Danvers, MA, USA), and anti-GAPDH (rabbit, 1:1000, Cell Signaling, Danvers, MA, USA) were used. Anti-rabbit/mouse HRP-conjugated antibody (1:10000, Abcam, Cambridge, UK) was used and the chemiluminescence signal detected using ChemiDoc (Bio-Rad, Hercules, CA, USA) and Quantity One software (Bio-Rad, Hercules, CA, USA) to quantify the signal intensity of different bands. Relative phosphorylated-p38 (p-p38), p-ERK, p-Akt, p-JNK, and p-NFκB expression was estimated upon normalization to their respective unphosphorylated protein (p38, ERK, AKT, JNK and NFκB).

### 2.6. Statistical Analysis

All quantitative data are expressed as means ± SD. The statistical analysis of the results was performed using one-way analysis of variance (ANOVA) with Dunnett’s post-test and two-tailed t test where applicable. Statistical significance was set as *p* < 0.05 (*), *p* < 0.01 (**), and *p* < 0.001 (***). Statistical analysis was done using Prism 7 (GraphPad, San Diego, CA, USA). Each experiment was repeated at least three times and representative experiments are shown.

## 3. Results

### 3.1. Irisin Promoted GAG Production by OA Chondrocytes

**Figure 1:**
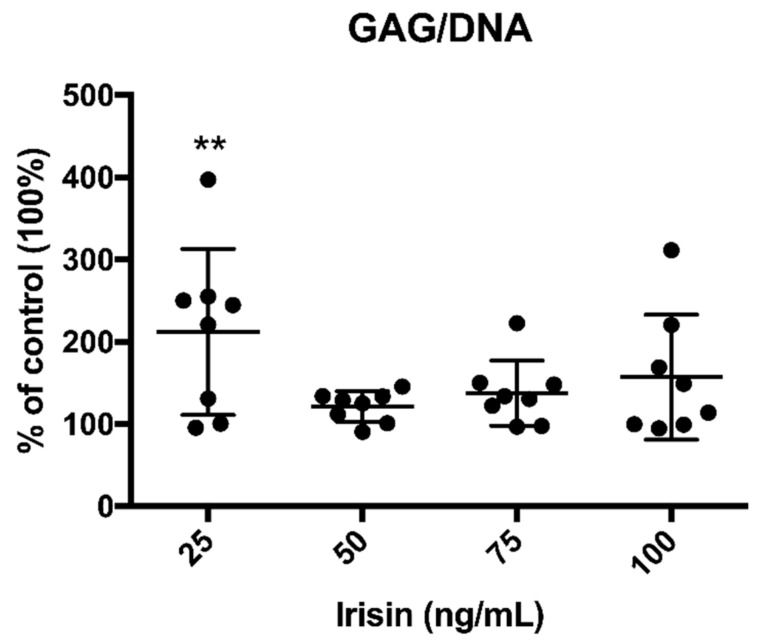
Irisin increases glycosaminoglycan (GAG) content in treated human osteoarthritic chondrocytes (hOAC). GAG/DNA content in hOAC after irisin treatment demonstrated a significant increase in the experimental group treated with 25 ng/mL. *n* = 8, **p < 0.01 compared to the control group.

Three-dimensional cell cultures (*n* = 8) treated with different concentrations of r-irisin (25, 50, 75 and 100 ng/mL) showed a significant increase in GAG synthesis normalized to DNA compared to the control cell cultures (Figure 1) at the lowest concentration (25 ng/mL). Considering the GAG/DNA ratio in the control group as a baseline of 100%, hOAC exposed to 25 ng/mL irisin showed approximately a 2-fold increase of GAG/DNA ratio (211.99 ± 100.45%; *p* < 0.01). Although showing an increase in GAG content, treatment with higher doses of r-irisin did not reach statistical significance (121.36% ± 18.54, 137.69 ± 39.72%, 157.22 ± 75.97% corresponding to 50, 75, and 100 ng/mL r-irisin, respectively).

### 3.2. Irisin Enhanced hOAC Numerosity

**Figure 2:**
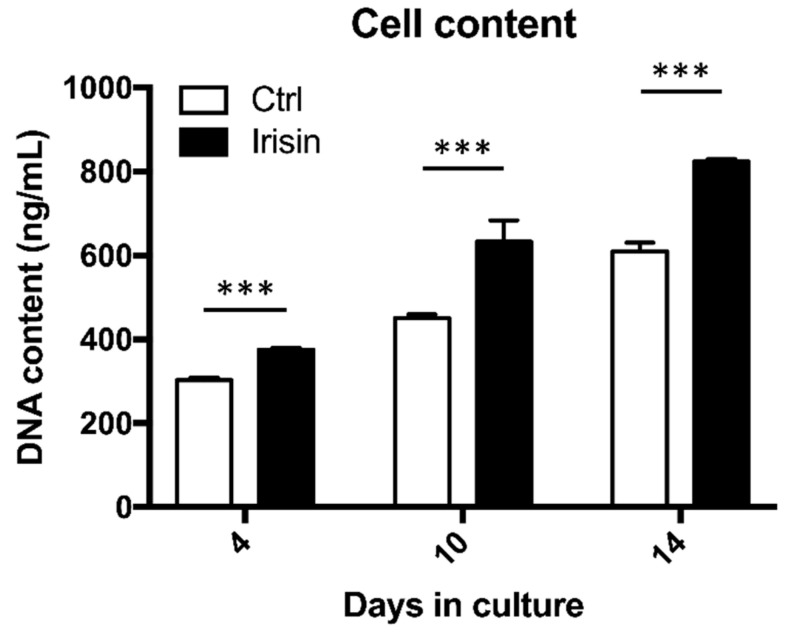
Irisin increases hOAC content. DNA concentration after treatment with 25 ng/mL irisin at day 0, 4, 10, and 14, as compared with the control group. *n* = 5, ***p < 0.001 compared to the control group at each timepoint. Ctrl, control group.

Treating hOAC with 25 ng/mL r-irisin resulted in a significant increase in cell content at 4, 10, and 14 days after starting three-dimensional cell culture (*n* = 5; Figure 2). At day 4, exposure to r-irisin led to a 12% (375.55 ± 4.49 ng/mL DNA) increase in cell content compared to the control group (303.2 ± 5.88 ng/mL DNA; *p* < 0.001). After 10 days of r-irisin treatment, the experimental group contained 633.08 ± 50.93 ng/mL DNA while control hOAC cultured with DPBS had 450.1 ± 9.86 ng/mL DNA (*p* < 0.001). At day 14, the mean hOAC DNA content after r-irisin exposure still remained significantly higher (823.2 ± 7.43 ng/mL DNA) in comparison to the control group (610.71 ± 7.43 ng/mL DNA; *p* < 0.001).

### 3.3. Irisin Restored the Normal ECM Gene Expression Profile of hOAC

**Figure 3:**
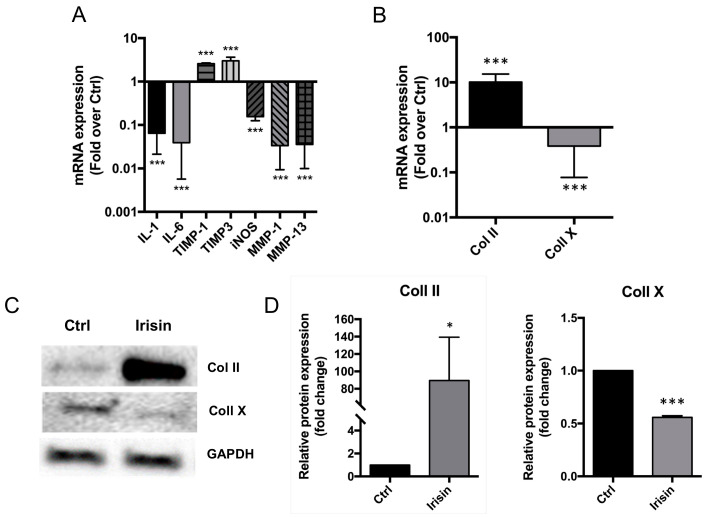
Irisin restores extracellular matrix (ECM) composition. (**A**) Irisin treatment significantly decreased IL-1, IL-6, iNOS, MMP-1 and MMP-13 gene expression, while increasing TIMP-1 and TIMP-3 mRNA levels. *n* = 3. (**B**) Type II collagen relative mRNA expression was significantly higher after 7 days of irisin exposure, as compared with the control group. Conversely, type X collagen relative mRNA expression was diminished upon irisin treatment compared to the control group. *n* = 3. (**C**) Western blot analysis confirmed the same trends, as type II collagen levels resulted higher whereas type X collagen levels were lower after irisin exposure at 7 days. *n* = 3. (**D**) Densitometric analysis of protein bands attested that these findings were statistically significant: type II collagen relative protein expression was increased (left chart), while type X collagen relative protein expression resulted to be lower at both intervals. Results were normalized based on GAPDH expression and calculated as fold change compared to the controls. *p = 0.037; ***p < 0.001. Ctrl, control group. IL, interleukin. TIMP, tissue inhibitor of metalloproteinases. iNOS, inducible nitric oxide synthase. MMP, matrix metalloproteinase. Coll II, collagen type II. Coll X, collagen type X. GAPDH, glyceraldehyde 3-phosphate dehydrogenase.

Irisin treatment exerted a beneficial effect on inflammatory and catabolic marker gene expression within hOAC (*n* = 3). Indeed, the experimental group showed a significant decrease of IL-1 (0.852 ± 0.008; *p* < 0.001), IL-6 (0.518 ± 0.025; *p* < 0.001); iNOS (0.164 ± 0.03; *p* < 0.001), MMP-1 (0.044 ± 0.011; *p* < 0.001), and MMP-13 (0.047 ± 0.012; *p* < 0.001) mRNA expression compared to the control group. Conversely, TIMP-1 (2.613 ± 0.019; *p* < 0.001) and TIMP-3 (2.871 ± 0.659; *p* < 0.001) gene expression were significantly incremented when compared to the controls (Figure 3A). After irisin exposure, mRNA expression of type II collagen underwent a substantial increase: the relative mRNA expression level of this gene was 10.08 ± 5.224 in the experimental group compared to controls (*n* = 8; *p* < 0.001). We also found a significant decreased mRNA expression of the hypertrophic chondrocyte-related gene encoding type X collagen (Figure 3B): the mRNA expression level was 0.384 ± 0.307 in the r-irisin group compared to the control group (*p* < 0.001). We confirmed these data by quantifying the gene product synthesis using Western Blot (Figure 3C). Irisin increased the protein levels of type II collagen and decreased the levels of type X collagen after 7 days of exposure (*n* = 3). These results were confirmed by densitometric analysis of protein bands (Figure 3D). Indeed, relative type II collagen expression after r-irisin exposure was 89.51 ± 49.89 times higher in the control group (*p* = 0.037). Conversely, relative type X collagen expression was 0.55 ± 0.01 compared to the control group following r-irisin treatment after 7 days (*p* < 0.001).

### 3.4. Irisin Mitigates OA-Related Changes via the p38 MAPK, Akt, and JNK Signaling Pathways

**Figure 4:**
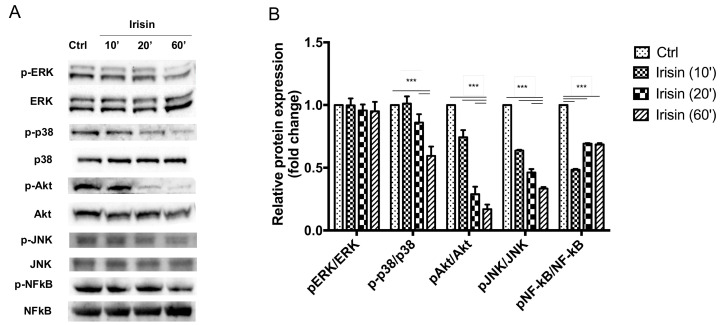
Irisin inactivates p38 mitogen-activated protein kinase (p38 MAPK), protein kinase B (Akt), c-Jun N-terminal kinase (JNK), and nuclear factor kappa-light-chain-enhancer of activated B cells (NFκB) pathways within hOAC. (**A**) Western blot analysis showed a reduction in p-p38, p-ERK, p-Akt, p-JNK and p-NFκB levels after irisin exposure at 10, 20, and 60 min. *n* = 5. (**B**) Densitometric analysis of protein bands demonstrated a significant decrease of p-p38 levels at 20 and 60 min. Similarly, p-Akt, p-JNK, and p-NFκB levels were significantly decreased at each time point. The p-ERK levels were not significantly decremented at each time point. *n* = 5. ***p < 0.001.

A decreased amount of p-p38, p-Akt, p-JNK and p-NFκB) in hOAC was detected by Western Blot from 10, 20, and 60 min after treatment with r-irisin (*n* = 5; Figure 4A). The decreased phosphorylation of p38, Akt, JNK and NFκB was statistically significant, as confirmed by densitometry (Figure 4B). The p-p38 relative protein expression was 1.011 ± 0.058 at 10 min, 0.86 ± 0.066 at 20 min, and 0.594 ± 0.074 after 60 min of r-irisin exposure (*p* < 0.001) compared to controls. Regarding p-Akt, relative protein expression was 0.743 ± 0.056 at 10 min (*p* < 0.001), 0.289 ± 0.058 after 20 min (*p* < 0.001) and 0.169 ± 0.036 at 60 min (*p* < 0.001) compared to the control group. The p-JNK relative protein expression levels were 0.637 ± 0.006 at 10 min (*p* < 0.001), 0.462 ± 0.027 at 20 min (*p* < 0.001) and 0.334 ± 0.012 after 60 min (*p* < 0.001) compared to controls. Concerning p-NFκB, protein levels were 0.483 ± 0.006 at 10 min (*p* < 0.001), 0.692 ± 0.004 at 20 min (*p* < 0.001) and 0.685 ± 0.01 after 60 min (*p* < 0.001) when compared to the control group. Conversely, p-ERK protein levels did not show significant changes.

## 4. Discussion

In this study, we report for the first time that irisin may directly target hOAC and promote cell proliferation and anabolism by increasing GAG and type II collagen synthesis along with TIMP-1 and -3 levels, while reducing expression of type X collagen as well as IL-1 and -6, MMP-1 and -13 and iNOS through inactivation of p38 MAPK, Akt, JNK, and NFκB signaling pathways. This is the first study showing that irisin can directly act on chondrocytes and attenuate OA-related cartilage degeneration in vitro, suggesting the existence of a cross-talk mechanism between muscle and cartilage.

Irisin is secreted by skeletal muscle in response to physical exercise and may theoretically promote chondrocyte anabolism so that cartilage can better adapt to increased load and friction during prolonged exercise. While irisin first reported effect was to promote adipocyte transdifferentiation and energy metabolism [26], irisin-induced proliferation, differentiation and anabolic effects were also observed with other cell types, including osteoblasts [27], bone marrow stromal cells [28], and human umbilical vein endothelial cells [29]. Recent research efforts have described the wide biological activity of such myokine, whose effects are pleiotropically exerted on several organs, namely the brain [30], the pancreas [31], the liver [15], the bone [28] and the skeletal muscle [32]. Our data expands the knowledge base for irisin, reporting its role in promoting chondrocyte anabolism.

We tested the anabolic effects of irisin by treating primary hOAC in a three-dimensional culture system with r-irisin for 7 days. As irisin effect on articular chondrocytes has not been reported before, we performed a dose-response experiment to assess the most effective concentration on GAG synthesis by using increasing doses within a range including intraarticular [22] and serum irisin concentration [19,25] as reported by previous studies. Our results showed that irisin increased the expression of type II collagen while reducing the expression of type X collagen, a marker of chondrocyte hypertrophy in osteoarthritic cartilage. In addition, we demonstrated that irisin was able to increase hOAC proliferation at all considered timepoints by disabling p38, Akt, JNK, and NFκB pathways which play a role in cartilage anabolic as well as catabolic processes in response to the activation of inflammatory processes of various origins. This has been demonstrated by irisin capacity to significantly reduce the expression of IL-1, IL-6, MMP-1, MMP-13, and iNOS, which have been described as key contributors to OA-related cartilage destruction in numerous studies [5,33]. The lower number of chondrocytes within osteoarthritic cartilage reduces the capacity of the tissue to counteract exogenous stresses and to maintain the original ECM composition. In this regard, increasing chondrocyte proliferation and TIMP-1 and -3 expression would enhance cartilage metabolism and capacity to react to stressful stimuli.

Our findings have demonstrated that irisin may reverse hOAC imbalance in anabolic and catabolic functions by inhibiting the phosphorylation of p38, Akt, JNK, and NFκB, thus suggesting that these signaling pathways may play a critical role in the chondrogenic effect of irisin.

The p38 and ERK signaling pathways are crucial to cell proliferation and differentiation [34] and may be the main pathways mediating irisin effects. Indeed, irisin can stimulate browning of white adipocytes through p38 and ERK MAPK [35], promotes human umbilical vein endothelial cell proliferation through the ERK signaling pathway [36] and osteoblast proliferation and differentiation via activating the phosphorylation of p38 and ERK [19]. Moreover, these pathways have been directly implied in OA pathogenesis [37]. In osteoarthritic cartilage, excessive amounts of basic fibroblast growth factor are released upon mechanical loading and activate several transduction pathways involving different MAPK, including ERK and p38. This ultimately leads to upregulation of metalloproteinases, namely ADAMTS-5, MMP-1 and MMP-13, resulting in type II collagen degradation and aggrecan fragmentation [38]. Furthermore, p38 seems to be involved in promoting chondrocyte hypertrophy and apoptosis, inhibiting cartilage synthesis and downregulating chondrocyte autophagy [39]. 

Extensive studies have revealed the function of Akt pathway in chondrocytes during endochondral ossification. Deletion of Akt1 results in delayed calcification [40], while Akt activation in embryonic chondrocytes promotes chondrocyte proliferation and inhibits hypertrophic differentiation [41]. However, the in vivo function of Akt signaling in the maintenance of articular cartilage homeostasis and in OA development is largely undefined, with different in vitro studies reporting contradictory results. PI3K/Akt signaling has been shown to play a chondroprotective role by regulating chondrocyte survival, proliferation and extracellular matrix synthesis [42,43]. In contrast, some studies have reported a detrimental effect of PI3K/Akt pathway on OA, which might be achieved through transduction of procatabolic stimuli or inhibition of articular chondrocyte autophagy [44,45]. Our findings demonstrated that increased cell content and ECM anabolism in chondrocytes treated with irisin were associated with a reduction of p-Akt and thus with a downregulation of the PI3K/Akt pathway. This is consistent with previous data regarding PI3K/Akt pathway involvement in irisin signaling on distinct cell types [46], although other studies reported increased levels of p-Akt [47]. Contrariwise, no significant change in ERK activity was recorded in this study. This suggest that ERK and PI3K/Akt role in irisin signaling is probably cell-specific and conditioned by local stimuli. Thence, further studies are needed to delineate the precise role of the PI3K/Akt pathway in hOAC.

JNK and NFκB signaling pathways have also been implicated in OA [48]. In hOAC, JNK is mainly activated by proinflammatory cytokines such as IL-1 and tumor necrosis factor (TNF)-α. After phosphorylation, JNK upregulates several downstream factors that ultimately enhance the synthesis of MMP-13, decrease proteoglycan synthesis and upregulates the production of IL-1 and TNF-α, thence creating a sort of catabolic positive feedback [49]. In chondrocytes, downstream activation of the NFκB transcriptional factor upregulates the expression of several catabolic markers, including MMP-1, MMP-13, iNOS, IL-1, IL-6, and TNF-α [33,50]. Therefore, irisin-mediated inactivation of such pathways may reasonably reduce cartilage catabolism by downregulating such degradation markers, hence increasing cell anabolism. This assumption is supported by a recent study from Li et al. [51], who evaluated irisin effect on a chondrosarcoma cell line and demonstrated that the myokine reversed the decrease of type II collagen and the increase of MMP-13 following IL-1 pretreatment and was associated with a downregulation of both Wnt/β-catenin and NFκB pathways. 

Physical training yields recognized benefits in preserving joints health and is one of the main conservative approaches for preventing and treating OA [52]. Exercise, by strengthening periarticular muscles along with general aerobic conditioning can improve joint stability, reduce pain and ameliorate quality of life [11,13]. Moreover, the administration of physiological dynamic loads, as during physical exercise, enhances the production of ECM components, including collagens, proteoglycans and oligomers by articular chondrocytes [53]. Conversely, disuse and limited movement due to severe illness, cachexia and muscular diseases can favor joint degeneration and rigidity [54]. However, the effect of physical training on both joint health and irisin serum concentration strictly depends on the type of exercise [55]. Several past studies have reported that resistance, anaerobic and high intensity exercise can increase irisin levels in the bloodstream [56,57], while aerobic exercise and reduced load training do not significantly influence irisin concentration [58]. Duration of exercise training and environmental factors both influence the levels of circulating irisin. A large meta-analysis reported a decrease of circulating irisin in healthy individuals undergoing either endurance or resistance chronic exercise (>8 weeks) [59], whilst another study showed a reduction in irisin levels after two weeks of climbing at high altitude-hypoxia [60]. To date, no evidence concerning the ideal type of exercise or training protocol for osteoarthritic joints is available. A large meta-analysis comparing high-intensity versus low-intensity exercise for knee and hip OA was inconclusive [61], although it is widely accepted that improving muscle strength, aerobic capacity and lowering body weight benefits joint maintenance and cardiovascular health [8].

The major limitation of this study is that results have been obtained through an in vitro experimental design, even though human primary cells have been used. Currently, no reports are available correlating irisin synovial fluid concentration with the type of physical activity performed in either healthy subjects or patients with OA. Therefore, these data need to be further confirmed in an experimental animal model of OA exposed to physical exercise. In addition, as the effect of irisin on articular chondrocytes under physiological conditions has not been described yet, our understanding of its biological role on hOAC might not encompass all the effects that the myokine would have on the healthy tissue. For this reason, additional studies comparing healthy chondrocytes with hOAC are needed to fully establish irisin physiological effect on cartilage. A further consideration limiting this study is related to the posttranslational glycosylation of irisin after secretion that enhances its biological function [35]. Indeed, most of commercial r-irisin derived from Escherichia Coli, including the one used in this study, is non-glycosylated. Therefore, the biological activity may not exactly reflect the myokine action on chondrocytes in vivo, which may be even stronger.

## 5. Conclusions

Our results indicate that irisin may be one of the mediators by which physical exercise and muscle tissues modulate cartilage metabolism, demonstrating the existence of a biological cross-talk mechanism between muscle and cartilage. Taken together, our data demonstrate the role of irisin in osteoarthritic chondrocyte metabolism and suggest that irisin can be used as a cartilage-regulating factor, which directly targets chondrocytes and enhances cell anabolism while decreasing catabolism, suggesting a potential therapeutic role in treating OA.

## Figures and Tables

**Table 1:** Patient age, sex and Kellgren–Lawrence (K-L) grades based on X-ray imaging.

Patient No.	Age (y)	Sex	K-L Stage
1	79	F	4
2	58	M	4
3	67	F	3
4	85	F	4
5	72	M	4
6	72	F	4
7	73	M	3
8	49	M	4

## Abbreviations

ADAMTS: A Disintegrin and Metalloproteinase with Thrombospondin motifs; Akt: protein kinase B; Ctrl: control; DPBS: Dulbecco’s phosphate-buffered saline; ECM: extracellular matrix; ERK: extracellular signal-regulated kinase; GAG: glycosaminoglycan; GAPDH: glyceraldehyde-3-phosphate dehydrogenase; hOAC: human osteoarthritic chondrocytes; IL: interleukin; iNOS: inducible nitric oxide synthase; JNK: c-Jun N-terminal kinase; MAPK: mitogen-activated protein kinase; MMP: matrix metalloproteinase; NFκB: nuclear factor kappa-light-chain-enhancer of activated B cells; OA: osteoarthritis; p-Akt: phosphorylated Akt; p-ERK: phosphorylated ERK; p-JNK: phosphorylated JNK; p-NFκB: phosphorylated- NFκB; p-p38: phosphorylated p38; PCR: polymerase chain reaction; r-irisin: recombinant irisin; TIMP: tissue inhibitor of metalloproteinases; TNF: tumor necrosis factor.
